# New chrysilline and aelurilline jumping spiders from Pakistan (Araneae, Salticidae)

**DOI:** 10.3897/zookeys.783.21985

**Published:** 2018-08-30

**Authors:** Pir Asmat Ali, Wayne P. Maddison, Muhammad Zahid, Abida Butt

**Affiliations:** 1 Department of Zoology, University of British Columbia, Vancouver, V6T 1Z4, Canada; 2 Department of Zoology, Islamia College University, Peshawar, Pakistan; 3 Department of Botany and Beaty Biodiversity Museum, University of British Columbia, Vancouver, V6T 1Z4, Canada; 4 Department of Zoology, Women University Swabi, Swabi, Pakistan; 5 Department of Zoology, University Of The Punjab, Lahore, Pakistan

**Keywords:** Aelurillina, Araneae, Chrysillini, *
Epocilla
*, Khyber Pakhtunkhwa, new species, Pakistan, Salticidae, *
Stenaelurillus
*

## Abstract

*Epocillapakhtunkhwa* Ali & Maddison, **sp. n.** and *Stenaelurillusmardanicus* Ali & Maddison, **sp. n.** are described from Khyber Pakhtunkhwa Province, Pakistan. Noted for the first time is the presence in *Epocilla* of a small bump just anterior to the fovea of the carapace, of unknown functional significance, otherwise known in the unrelated *Opisthoncus* L. Koch, 1880 and *Cocalus* Pocock, 1897. In addition, the female of *Menemerusnigli* Wesołowska & Freudenschuss, 2012 is described for the first time.

## Introduction

The salticid spider fauna of Pakistan is little studied, with only a few species reported to date (Azarkina 2004; Bauer et al. 2015; Logunov and Azarkina 2018; Logunov et al. 2011; Wesołowska and Freudenschuss 2012). However, an ongoing project to discover the country’s salticid diversity is revealing that many species are present, especially of the tribes Chrysillini and Aelurillini. In this paper two of the species discovered in this project are described, a chrysilline of the genus *Epocilla* Thorell, 1887, and an aelurilline of the genus *Stenaelurillus* Simon, 1886. For the first time, the female of *Menemerusnigli* Wesołowska & Freudenschuss, 2012 is also described. The species described here highlight the mixed fauna of Pakistan, holding the eastern extensions of primarily African groups (like *Menemerus*), and the western extensions of primarily Asian Groups (like *Epocilla*).

The descriptions presented here are part of a series of works that are the first to focus specifically on collecting and documenting the Salticidae of Pakistan (Ali et al. 2016). This accompanies the ongoing work in neighbouring India (Caleb 2016, 2017; Caleb and Mathai 2015, 2017; Caleb et al. 2015, 2017a, 2017b; Chatterjee et al. 2017; Kananbala et al. 2014; Prajapati et al. 2016; Sanap et al. 2017; Sebastian et al. 2015; Vidhel et al. 2015) in building our knowledge of South Asian salticids.

## Material and methods

Preserved specimens were examined under both dissecting microscopes and a compound microscope with reflected light. Photographs of bodies in alcohol were taken either with a Leica MZ 16 or with a Nikon 745 microscope. Male palps and female epigyna were removed for study and placed in a depression slide, examined with incident light on a Nikon ME600L compound microscope. Drawings were made with a drawing tube on this compound microscope. For cleared images, epigyna were suffused with clove oil for a few minutes. Laboratory work was carried out in the Maddison Lab, University of British Columbia, Canada and in the Department of Entomology, Agriculture University Peshawar, Pakistan.

Measurements are given in millimeters. Carapace length was measured from the base of the anterior median eyes not including the lenses to the rear margin of the carapace medially; carapace width measured as maximum width; carapace height is maximum from lateral view. Abdomen length measured to the end of anal tubercle, not including spinnerets; width is the maximum. Ocular area and eye row measurements include lenses of relevant eyes. Abbrevations:

**AME** Anterior median eyes,

**ALE** Anterior lateral eyes,

**PLE** Posterior lateral eyes,

**PME** posterior median eyes,

**AER** Anterior eye row,

**RTA** retrolateral tibial apophysis.

Until a fully curated natural history museum is established in Pakistan, the specimens will be held in the Spencer Entomological Collection at the Beaty Biodiversity Museum, University of British Columbia (**UBC**).

## Taxonomy

### Tribe Chrysillini Simon, 1901

The Chrysillini includes about 600 species in 31 genera (Maddison 2015) of which *Epocilla* Thorell, 1887 and *Menemerus* Simon, 1868 are unusual for their large bodies and robust legs. *Epocilla* is especially distinctive for its robust first legs and a striking appearance of orange stripes. The genus includes eleven described species, restricted to south and southeast Asia except for one species in Mauritius and one reaching Hawaii (Peng et al. 1993; Saaristo 2002; Jastrzębski 2007; Caleb et al. 2017a; World Spider Catalog 2018). The westernmost localities in mainland Asia reported to date are in India, but recent collecting in Pakistan has revealed a new species, which we describe here as *E.pakhtunkhwa*, new species. Our collecting also allows us to describe for the first time the female of *Menemerusnigli* Wesołowska & Freudenschuss, 2012.

#### *Epocilla* Thorell 1887

Described by Thorell (1887) with *E.praetextata* as type species, *Epocilla* is distinctive among chrysillines for the long body with robust legs. Males are strongly built, with longitudinal orange streaks. The male palp has a double retrolateral tibial apophysis (Żabka 1985), consisting of a flat rounded projection, dorsal and prolateral to which is a more normal, pointed apophysis. On the retrolateral surface of the tegulum is a distinct bump, typical of chrysillines (Figure 3, "B"). The embolus varies in length and is sometimes sinuous (Żabka, 1985). As noted above, the genus is primarily south and southeast Asian. *Epocillaaurantiaca* (Simon, 1885), *E.chimakothiensis* Jastrzebski, 2007, *E.mauriciana* Simon, 1901, *E.praetextata* Thorell, 1887, *E.sirohi* Caleb, Chatterjee, Tyagi, Kundu & Kumar, 2017, and *E.xylina* Simon, 1906 are distributed in India, Bhutan, Mauritius and Sri Lanka of south Asia. *Epocillablairei* Żabka, 1985, *E.calcarata* (Karsch, 1880), *E.femoralis* Simon, 1901, *E.innotata* Thorell, 1895 and *E.picturata* Simon, 1901 are distributed in southeast Asia. *Epocillapraetextata* Thorell, 1887, *E.calcarata* (Karsch, 1880) and *E.aurantiaca* (Simon, 1885) occur in both regions.

**Figures 1–4.**
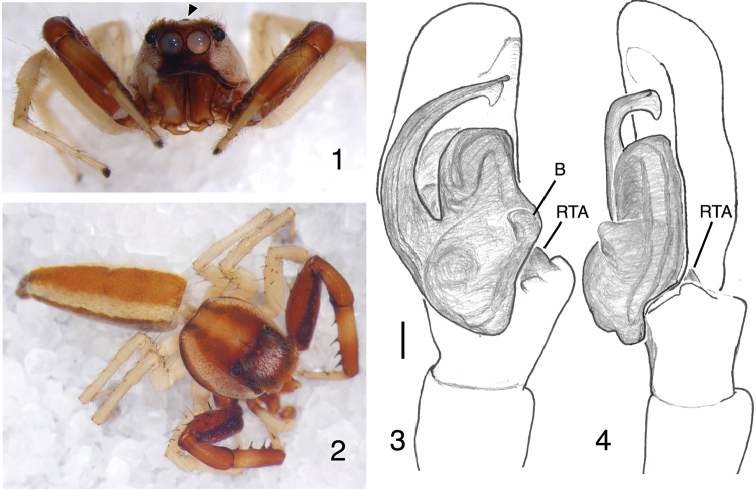
*Epocillapakhtunkhwa* Ali & Maddison, sp. n., male holotype (PAA#2015-07-146). **1** face **2** dorsal view **3** left palp, ventral view **4** left palp, retrolateral view. Scale bar: 0.1 mm (**3, 4**). Abbreviations: B = bump on tegulum, RTA = retrolateral tibial apophysis. Figures 1–4 are copyright ©2017 Pir Asmat Ali and Wayne Maddison, released under a Creative Commons Attribution (CC-BY) 3.0 license.

An unusual feature of some species of *Epocilla* is the presence, in both males and females, of a distinct integumental bump in the ocular area, in front of the fovea and between the PLE (Figure 1, triangle; visible on Figure 2 as a small paler spot on the midline between the anterior edges of the PLE). Although not reported previously in *Epocilla*, we have observed it in both males and females of *Epocillacalcarata* from Sarawak, Malaysia. It can also be seen in figures in the literature, though without comment. Prószyński (1987, p. 26) shows it in a drawing of a paralectotype of *E.aurantiaca*, and Kananbala et al. (2014, figure 2) show it in a photograph of *E.praetextata*. A similar bump just anterior to the fovea is well known in the astioid *Opisthoncus* (e.g., Gardzińska and Żabka 2013) and the spartaeine *Cocalus* (Wanless, 1981). Its functional significance, if any, is unclear. The fact that it is present in females in all three genera suggests that it is not a courtship ornament. As it is distinctly in front of the fovea, it is presumably not an attachment point of the lorum-dorsal apodeme muscle. It is approximately where one set of eye muscles would attach to the carapace.

##### Epocilla pakhtunkhwa

Taxon classificationAnimaliaEpocillaSalticidae

Ali & Maddison sp. n.

http://zoobank.org/0EDF9267-D695-4E1F-933A-3C32C508495B

Figs 1–4

###### Holotype.

Male in UBC from Pakistan: Khyber Paktunkhwa: Malakand (Agra), 34.589°N, 71.713°E, 2500 m elevation, 7 July 2015, Pir Asmat Ali, from maize crop fields (specimen PAA#2015-07-146).

###### Etymology.

The name of the province of the type locality, treated as a noun in apposition.

###### Diagnosis.

*Epocillapakhtunkhwa* is most similar to *E.sirohi* (Caleb et al. 2017a) in having a long embolus curving retrolaterally, but differs in having an expanded flange near the tip of the embolus (lacking in *E.sirohi*; Caleb et al. 2017a) and a narrower and deeper cleft between the embolus and tegulum. Two other species with a long embolus are *E.aurantiaca* and *E.blairei*, but these have an embolus that is sinuous, curving toward the retrolateral but then reversing the curve to point toward the tip of the cymbium (Prószyński 1984; Żabka 1985).

###### Description.

**Male** (holotype): *Measurements*. Carapace length 2.63, width 2.35, height 1.50. Ocular area widest at AER; length 1.31, width of PLE row 1.54, width of PME row 1.50, AER width 1.59. Abdomen length 3.95, width 1.16. Leg I: coxa 0.94, trochanter 0.56, femur 2.06, patella 1.41, tibia 1.69, metatarsus 1.50, tarsus 0.56. Leg II: coxa 0.75, trochanter 0.27, femur 1.88, patella, 0.86; tibia, 1.69; metatarsus, 1.43, tarsus, 0.56. Leg III: coxa 0.75, trochanter 0.37, femur 1.88, patella 0.84, tibia 1.22, metatarsus 1.41, tarsus 0.75. Leg IV: coxa 0.93, trochanter 0.47, femur 1.97, patella 0.84, tibia 1.50, metatarsus 1.41, tarsus 0.75.

*Structure*. Ocular area with a distinct integumental bump in front of the fovea and between the PLE (Figure 1, triangle). Carapace sides extend laterally with gentle slope in thoracic and cephalic regions, making the thoracic area broad. Posterior to fovea, thoracic area slopes gently before sloping more abruptly near back margin. Chelicera with one simple retromarginal tooth and 2 promarginal teeth; basal segment more or less vertical, relatively long, narrowing toward the tip but then expanding just before the fang (Figure 1). Palp tibia swollen retrolaterally, with dual apophysis as typical of *Epocilla*. The more prolateral of these is the RTA itself, somewhat hidden (Figs 3, 4, "RTA"). End of cymbium fairly wide, blunt. Embolus long, with an expanded flange near the tip, though the tip itself is narrow and curves slightly toward the ventral. Leg I stout, with tibia ventrally having 4 pairs of macrosetae, the anterior of each of which is much larger. Abdomen squared at the anterior margin, somewhat cone-shaped to the posterior.

*Colour*. Carapace edges are dark brown, above which are broad lateral bands of yellow white scales extending from beneath the ALE on the clypeus to the posterior edge and lying over orange-brown integument. Centrally the thoracic region is reddish brown with a medial paler area, with traces of brown hairs and scales present. Ocular area black to dark brown, with fringe of orange hairs at front extending over the AER, appearing from the front as two eyebrows. Posterior to that is a patch of white scales centrally in the ocular area, surrounded by darker scales. Clypeus dark brown below AME, having a few orange hairs, contrasting strongly with white scales under ALE that belong to the lateral carapace bands. Chelicerae yellowish brown near fangs, darker brown otherwise. Palp coxa and trochanter light brown; femur, patella and tibia paler orange-yellow; cymbium brown with orange-yellow tip. Sternum whitish yellow with edges brown. Leg I medium brown except for a much darker longitudinal brown band along the prolateral side of the coxa, trochanter and femur, a lighter patch on the tibia, and pale yellow-white metatarsus and tarsus. Legs II, III, IV pale yellow-white. Abdomen longitudinally striped, with wide brown band running medially, lateral to which is a band of yellow-white scales, lateral and ventral to which are reflective transparent or grey scales. Spinnerets are yellow-brown with grey hairs.

**Female**: unknown.

###### Habitat.

Specimens were collected by hand picking from maize, both living and cut, in maize fields.

###### Additional material examined.

One male paratype from Pakistan: Khyber Paktunkhwa: Malakand (Manzari Baba), 34.49°N, 71.71°E, 1613 m elevation, 13 July 2015, Pir Asmat Ali (specimen PAA#2015-07-134).

#### *Menemerus* Simon, 1868

*Menemerus* includes at present 67 nominal species (World Spider Catalog 2018), the majority distributed in Africa, the Middle East and the Arabian Peninsula (Wesołowska 1999; Prószyński 2003; Logunov 2010; Wesołowska & van Harten 2011; Wesołowska and Freudenschuss 2012), with five species from the Oriental Region (Jastrzębski 1997; World Spider Catalog 2018).

##### Menemerus nigli

Taxon classificationAnimaliaAraneaeSalticidae

Wesołowska & Freudenschuss, 2012

Figs 5–13

###### Notes.

This species was described by Wesołowska and Freudenschuss (2012) based on a male from Baluchistan Province, Pakistan, and redescribed (also from the male) by Chatterjee et al. (2017) from India. In our collecting, we found a male matching *M.nigli* in close proximity and in similar habitat to a female of similar size and markings. As no other species of *Menemerus* has yet been collected from Pakistan, except the distinctly different *M.bivittatus* (Dufour, 1831) and *M.marginatus* (Kroneberg, 1875) (Bauer et al. 2015), we interpret the female collected to be that of *M.nigli*. The female is described for the first time.

###### Description.

**Figures 5–13.**
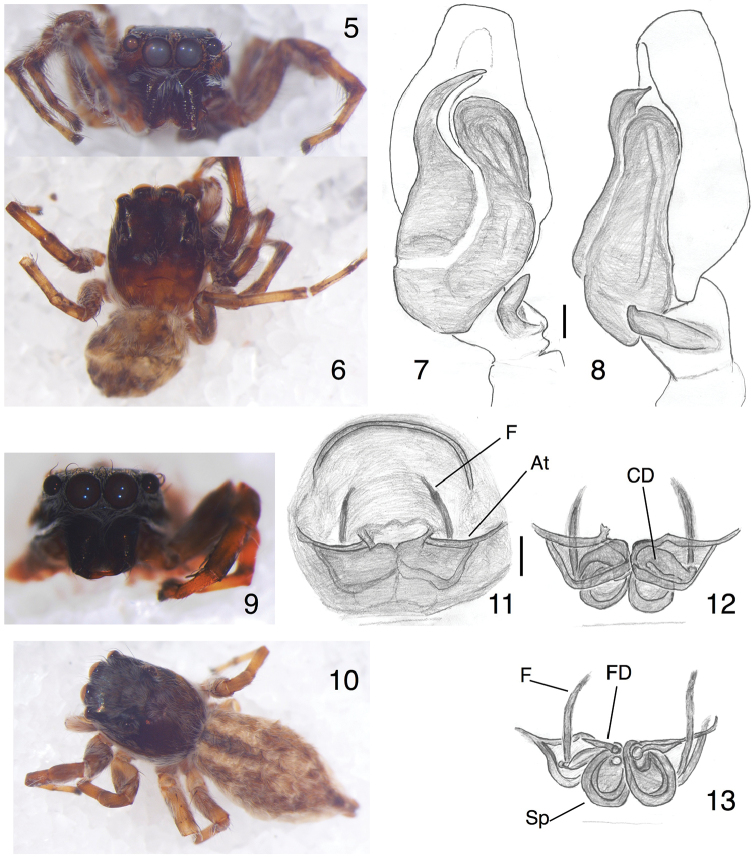
*Menemerusnigli*. **5–8** male (PAA#2015-07-081). **5** face **6** dorsal view **7** left palp, ventral view **8** left palp, retrolateral view. **9–13** female (PAA#2015-07-086): **9** face **10** dorsal view **11** epigynum, ventral view **12** epigynum cleared, ventral view **13** copulatory ducts and spermathecae, dorsal view. Scale bars: 0.1 mm (**7, 8, 10, 11**). At = Atrium, F = fold in wall, CD = copulatory duct, FD = fertilization duct, Sp = spermatheca. Figures 5–13 are copyright ©2017 Pir Asmat Ali and Wayne Maddison, released under a Creative Commons Attribution (CC-BY) 3.0 license.

**Male** (from Pakistan: Khyber Paktunkhwa: Karak (Karak city), 33.11°N, 71.08°E, elev. 550 m, 29 July 2015, Pir Asmat Ali, specimen PAA#2015-07-081). Carapace length 2.72, width 2.06, height 1.12. Abdomen length 2.35, width 2.06. Palp as in the figures of Wesołowska and Freudenschuss (2012). Embolus longer than in most *Menemerus* species, curved. A membranous "conductor" beside the embolus leads to a membranous area that forms a groove separating the embolus from the tegulum. Tibial apophysis elongate and fairly thin, pointing somewhat ventrally (Figs 7–8). Prolateral side of tibia swollen (Wesołowska and Freudenschuss 2012, figure 6). Cymbium with short white setae; femur with long brown and white hairs. Body and face as in Figs 5–6.

**Female** (from Pakistan: Khyber Paktunkhwa: Karak (Serekhawah), 33.27°N, 71.21°E, elev. 509 m., 29 July 2015, Pir Asmat Ali, specimen PAA#2015-07-086), Measurements: Carapace: length 2.82, width 2.06, height 1.31. Ocular area widest at AER; ocular area length 1.41, width of PLE row 1.59, width of PME row 1.50, AER width 1.76. Abdomen: length 3.76, width 2.16. Leg I: coxa 0.65, trochanter 0.47, femur 1.50, patella 0.84, tibia 1.03, metatarsus 0.75, tarsus 0.47. Leg II: coxa 0.56, trochanter 0.37, femur 1.31, patella 0.75, tibia 0.94, metatarsus 0.75, tarsus 0.37. Leg III: coxa 0.65, trochanter 0.37, femur 1.50, patella 1.08, tibia 1.22, metatarsus 0.47, tarsus 0.56. Leg IV: coxa 0.75, trochanter 0.37, femur 1.59, patella 0.84, tibia 1.41, metatarsus 1.12, tarsus 0.56.

*Structure*. Carapace flat in the cephalic region, dropping down to a lower thoracic area. Chelicerae stout, unidentate, with two promarginal teeth and long fangs. Posterior lateral spinnerets longer than others. Epigynum (Figs 11–13) with broad atria (Figure 11, "At") opening toward the anterior, on the inner (dorsal) wall of which is a sharp fold (Figure 11, "F"), which may serve to guide the entrance of the embolus. The precise point at which the atrium becomes narrowed into the copulatory duct is unclear, but it appears that the path of the embolus (or sperm) would be toward the posterior initially in the broad atrium, then turning medially, and once at the midline then turning posteriorly, at that point clearly in the copulatory duct. Spermatheca touching at midline, overlapped. Fertilization duct long.

*Colour* (Figs 9). Carapace brown, with cephalic sides dark brown covered by brown and white bristles and a basal band of white hairs. Ocular area black with brown and white bristles. Face black with short and long white bristles; chelicera almost black. Sternum yellow-brown. Legs brown to yellowish brown, metatarsus and tarsus palest; leg I darkest. Dorsum of abdomen with indistinct brown to cream markings, in the anterior half forming a darker longitudinal medial band near the base flanked by two whitish bands, and in the posterior half having a medial whitish patch. Venter is grey with scattered white hairs. Spinnerets yellow.

###### Remarks.

The female of *Menemerusnigli* can be distinguished from other *Menemerus* species by the broad forward-opening atria from which extend distinctive folds (Figure 11, "F"). There are other species with broad atria that open more or less to the anterior, but in most the atria are less deep and more laterally-facing: *Menemerusmarginatus* (Kroneberg, 1875) (see Bauer et al. 2015), *Menemerusminshullae* Wesołowska, 1999 (see Wesołowska 1999), and *Menemerusrubicundus* Lawrence, 1928 (see Wesołowska 1999). The epigynum of *Menemerusnamibicus* Wesołowska, 1999 is perhaps most similar to *M.nigli*, in that the atria face directly to the anterior, but they are not so spacious, and the distinctive fold of *M.nigli* is lacking (Wesołowska 1999).

### Tribe Aelurillini, Subtribe Aelurillina Simon 1901

The subtribe Aelurillina includes more than 260 species in nine genera in the Old World (Maddison 2015; Logunov and Azarkina 2018), and a single species in the New World (Logunov and Koponen 2002). *Stenaelurillus*, recently reviewed by Logunov and Azarkina (2018), are ground-dwelling spiders, notable for the highly ornamented males of some species (Caleb and Sanap 2016). Most of its 45 known species are African (World Spider Catalog 2018; Wesołowska 2014b; Logunov and Azarkina 2018), but there is a centre of diversity in south Asia (see figure 507 of Logunov and Azarkina 2018), with 8 species in India (Caleb et al. 2015; Caleb et al. 2017b; Caleb and Mathai 2016; Logunov and Azarkina 2018; Prajapati et al. 2016; Sebastian et al. 2015; Vidhel et al. 2015; Wesołowska 2014a), one from Iran (Logunov 2001), and one from Pakistan (Logunov and Azarkina 2018). We here describe a second species from Pakistan.

#### *Stenaelurillus* (Simon, 1885)

The genus *Stenaelurillus* contains medium-sized aelurillines with a moderately high carapace, widest posteriorly at coxae of third legs. Abdomen of distinctive shape, having the anterior edge straight and with long dense bristles, and a typical colour pattern composed of a paler transverse anterior band and three rounded spots posteriorly (except for *S.furcatus* Wesołowska, 2014, *S.nigricaudus* Simon, 1886, and *S.sudanicus* Wesołowska, 2014, which have two longitudinal lateral brown bands and one median white band). Legs III and IV longer than legs I and II. Embolus short.

##### Stenaelurillus mardanicus

Taxon classificationAnimaliaAraneaeSalticidae

Ali & Maddison sp. n.

http://zoobank.org/37D0BD67-21A4-4CF1-9674-194A55E933C8

Figs 14–21 , 22–26

###### Holotype.

Male in UBC from Pakistan: Khyber Paktunkhwa: Mardan (Sarmalang), 34.374°N, 72.372°E, 1443 m elevation, 10 August 2016, Pir Asmat Ali, foothills of mountains (specimen PAA#2016-08-101).

###### Etymology.

Derived from the name of the district of the type locality.

###### Diagnosis.

The embolus of *S.mardanicus* is distinctive (Figs 18, 19), with a broad basal portion curving dorsally toward the cymbium then prolaterally, and a short terminal piece (with the opening) pointing distally. The embolus appears therefore as a hand, with the terminal piece like a thumb sticking up, and the basal portion like curved fingers. In ventral view, the base of the embolus appears as an embolar ledge (EL) (Figs 18, 19, 20, 21). An embolus of this shape is unique among known *Stenaelurillus*. Two species with a similar prolateral extension near the base of the embolus are *S.triguttatus* Simon 1886 from Nepal, and *S.arambagensis* (Biswas & Biswas, 1992) from Pakistan, but in both of those species the extension points distally (Wesołowska 2014a; Logunov and Azarkina 2018) and at least in *S.arambagensis* it is not part of the embolus, but rather of the functional tegulum (Logunov and Azarkina 2018: 20). The prolateral extension of *S.mardanicus* is clearly part of the embolic division (Figs 18, 19, 20). *Stenaelurillusmarusiki* Logunov, 2001 also has an embolar ledge, but the embolus tip is much longer and narrower. *Stenaelurillusgabrieli* Prajapati, Murthappa, Sankaran & Sebastian, 2016 from India also has a short distally-pointing embolus tip, but lacks the embolar ledge as in *S.mardanicus*. The epigynum of *S.mardanicus* resembles that of the African *Stenaelurillusmirabilis* Wesołowska and Russell-Smith, 2000, with copulatory ducts relatively short, running from the small anteriorly-placed copulatory to join the spermathecae toward their posterior end (Figs 24, 25). *S.mirabilis* differs, however, in having a long pocket along the posterior margin (Wesołowska and Russell-Smith 2000).

**Figures 14–21.**
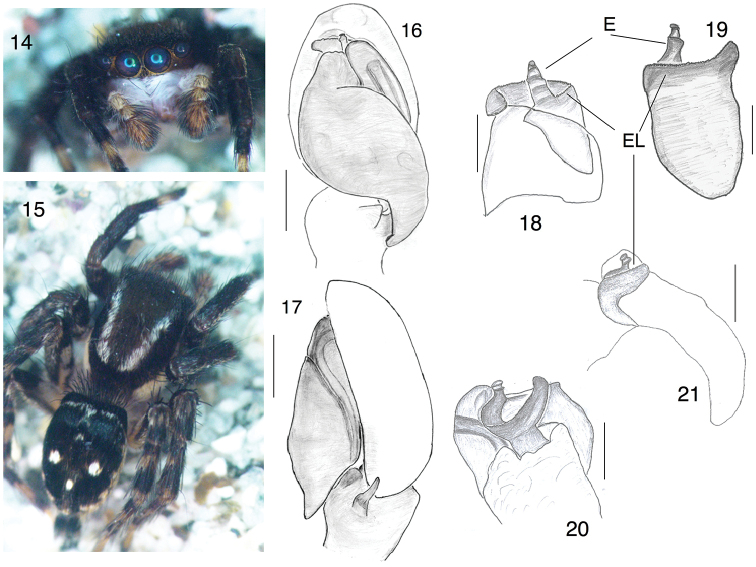
*Stenaelurillusmardanicus* sp. n., male paratype (specimen PAA#2015-07-154). **14** face **15** dorsal view **16** left palp, ventral view **17** left palp, retrolateral view **18** embolus of left palp, ventral view **19** embolus, dorsal view **20** embolus, retrolateral view **21** embolus, prolateral view. Scale bars: 0.1 mm. Abbreviations: E = embolus, El = embolar ledge. Figures 14–21 are copyright ©2017 Pir Asmat Ali and Wayne Maddison, released under a Creative Commons Attribution (CC-BY) 3.0 license.

**Figures 22–26.**
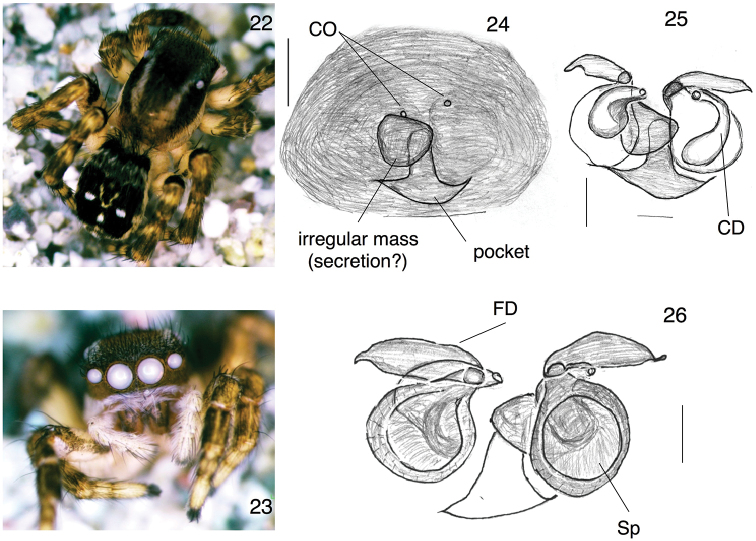
*Stenaelurillusmardanicus* sp. n., Female paratypes (**22** and **23** of specimen PAA#2016-08-120 **24–26** of specimen PAA#2015-07-114), **22** body, dorsal view **23** face **24** epigynum, dorsal view **25** cleared epigynum, ventral view **26** cleared epigynum, dorsal view. Scale bars: 0.1 mm. Abbreviations: CO = copulatory opening, CD = copulatory duct, FD = fertilization duct, Sp = spermatheca. Figures 22–26 are copyright ©2017 Pir Asmat Ali and Wayne Maddison, released under a Creative Commons Attribution (CC-BY) 3.0 license.

###### Description.

**Male** (holotype and paratype PAA#2015-07-154, Pakistan: Khyber Paktunkhwa: Malakand, Palai 34.5353°N, 72.0896°E 841 m elev. 12 July 2015 Pir Asmat Ali; measurements from that paratype): *Measurements*. Carapace length 2.50, width 1.97. height 1.10. ocular area widest at AER; AE length 1.61, PME width 1.40, PLE 1.55. Abdomen length 1.79, width 1.48. Leg I: coxa 0.35, trochanter 0.30, femur 1.17, patella 0.35, tibia 0.55, metatarsus 0.50, tarsus 0.43. Leg II: coxa 0.43, trochanter 0.30, femur 1.05, patella 0.55; tibia 0.63; metatarsus 0.60; tarsus 0.41. Leg III: coxa 0.49; trochanter 0.51, femur 1.50, patella 0.65, tibia 0.92, metatarsus 1.31, tarsus 0.65. Leg IV: coxa 0.90, trochanter 0.41, femur 1.77, patella 0.74, tibia 1.34, metatarsus 1.31, tarsus 0.65.

*Structure*. Carapace elevated at cephalic region, sloping gently down in thoracic region then abruptly before the posterior margin. Cheliceral retromargin with one long tooth; promargin with two teeth. Leg I short. Abdomen square at anterior, widest at middle and narrowing toward anal tubercle. Palp tibia swollen and having two apophyses, ventral one (somewhat hidden) and the RTA. Cymbium wide at middle. Embolus as described in diagnosis, with a basal curved portion and a short, distally-pointing tip.

*Colour*. Carapace brown with black hairs except for a broad bands laterally along lower margin that are paler and with white hairs, and narrow longitudinal bands dorsally of white hairs just medial to the PLE and extending from PME to the thorax; brown recumbent hairs with black bristles in the ocular area. Lateral white bands extend onto clypeus, which is pale and with a mix of long white and some brown hairs. Chelicerae pale with long white hairs. Sternum pale; labium pale brown and paler anteriorly; maxilla pale brown; palp yellow; femur with long white hairs, cymbium brown and having black bristles and scales. First leg is darkest, with whitish yellow tarsus. Other legs pale with dark stains and black hairs, except ventral coxa of leg III and IV which are pale whitish. Front of abdomen square, with long grey bristles. Abdomen black above, with sub-basal transverse band of white hairs, and with three whitish pale spots: a pair near the middle and a single smaller posterior spot. Sides of abdomen with scattered pale hairs and black hairs. Venter pale with grey hairs. Spinnerets yellow with grey hairs.

Female (paratype, specimen PAA#2015-07-114, Pakistan: Khyber Paktunkhwa: Mardan (Baroch), 34.381°N, 72.384°E, elev. 1021 m elevation, 21 July 2015, Pir Asmat Ali, mountain edges): Measurements: Carapace length 2.63, width 2.03, height 1.30. Ocular area length 0.84, width 1.50, PLE width 1.50, PME width 1.41, AE 1.59. Abdomen length 2.63, width 1.88. Leg I: coxa 0.47, trochanter 0.37, femur 1.41, patella 0.65, tibia 0.65, metatarsus 0.37, tarsus 0.47. Leg II: coxa 0.47, trochanter 0.37, femur 1.12, patella 0.65, tibia 0.65, metatarsus 0.57, tarsus 0.37. Leg III: coxa 0.75, trochanter 0.55, femur 1.78, patella 0.74, tibia 1.22, metatarsus 1.41, tarsus 0.75. Leg IV: coxa 0.93, trochanter 0.47, femur 1.97, patella 0.84, tibia 1.50, metatarsus 1.41, tarsus 0.75.

*Structure*. Carapace elevated at cephalic region, sloping laterally and posteriorly in thoracic region. Chelicera with one long retromarginal tooth; promargin with two teeth. Leg I strong and short. Abdomen square at anterior, widening toward the posterior, then narrowing toward anal tubercle. Epigynum (Figs 24, 25) with a central pocket displaced slightly forward from the epigastric furrow. Copulatory openings are small, anterior. Copulatory ducts proceed from the openings toward the posterior to join the spermathecae, widening as they go. Spermathecae wide and round; fertilization ducts long and wide.

*Colour*. Carapace marked similarly to male: brown, with broad bands laterally along lower margin that are paler and with white hairs, and narrow longitudinal bands dorsally of white hairs just inside the PLE and extending from PME to the thorax. Ocular area black with recumbent brown hairs and bristles. Lateral white bands become narrower as they extend onto clypeus, which is pale and with a mix of white and brown long hairs. Chelicerae pale, with many white hairs and a few scattered black ones. All legs pale with dark markings and black hairs except ventral coxae of leg III and IV which are pale. Sternum pale; labium brown; maxillae pale brown with paler tips. Front of abdomen square, with long grey bristles. Abdomen black with a pair of white spots just posterior to the middle. Sides with pale band extending to the spinnerets. Venter pale yellow with scattered grey hairs. Spinnerets yellow with grey hairs.

###### Remark.

All observed females had epigynal plugs.

###### Habitat.

Specimens were found on mountain edges.

###### Additional material examined.

The following, all paratypes: Same data as holotype (female specimen PAA#2016-08-120, and 3 additional males); Pakistan: Khyber Paktunkhwa: Manzari baba (Malakand), 34.51°N, 71.72°E, elev. 2013 m, 13 July 2015, Pir Asmat Ali, mountain edges (1 female specimen PAA#2015-07-165); Pakistan: Khyber Paktunkhwa: Mardan, 34.354°N, 72.382°E, elev. 1430 m, 10 Aug 2016, Pir Asmat Ali (10 males, 2 females, 2 juveniles); Pakistan: Khyber Paktunkhwa: Alizai (Hangu), 33.58°N, 71.28°E, elev. 1678 m, 27 July 2015, Pir Asmat Ali, foothills of mountains (female specimen PAA#2015-07-087).

## Supplementary Material

XML Treatment for Epocilla pakhtunkhwa

XML Treatment for Menemerus nigli

XML Treatment for Stenaelurillus mardanicus
